# Implantable Pulse Generators for Deep Brain Stimulation: Challenges, Complications, and Strategies for Practicality and Longevity

**DOI:** 10.3389/fnhum.2021.708481

**Published:** 2021-08-26

**Authors:** Can Sarica, Christian Iorio-Morin, David H. Aguirre-Padilla, Ahmed Najjar, Michelle Paff, Anton Fomenko, Kazuaki Yamamoto, Ajmal Zemmar, Nir Lipsman, George M. Ibrahim, Clement Hamani, Mojgan Hodaie, Andres M. Lozano, Renato P. Munhoz, Alfonso Fasano, Suneil K. Kalia

**Affiliations:** ^1^Division of Neurosurgery, Department of Surgery, University of Toronto, Toronto, ON, Canada; ^2^Division of Neurosurgery, Department of Surgery, Université de Sherbrooke, Sherbrooke, QC, Canada; ^3^Department of Neurology & Neurosurgery, Center Campus, Universidad de Chile, Santiago, Chile; ^4^Department of Surgery, College of Medicine, Taibah University, Almadinah Almunawwarah, Saudi Arabia; ^5^Department of Neurosurgery, University of California, Irvine, Irvine, CA, United States; ^6^Department of Neurosurgery, Henan University School of Medicine, Zhengzhou, China; ^7^Department of Neurosurgery, University of Louisville School of Medicine, Louisville, KY, United States; ^8^Harquail Centre for Neuromodulation, Sunnybrook Health Sciences Centre, Toronto, ON, Canada; ^9^Krembil Research Institute, University Health Network, Toronto, ON, Canada; ^10^CRANIA Center for Advancing Neurotechnological Innovation to Application, University of Toronto, ON, Canada; ^11^Edmond J. Safra Program in Parkinson’s Disease Morton and Gloria Shulman Movement Disorders Clinic, Toronto Western Hospital, and Division of Neurology, Toronto Western Hospital, University of Toronto, Toronto, ON, Canada; ^12^KITE, University Health Network, Toronto, ON, Canada

**Keywords:** battery life, neuromodulation, complications, DBS (deep brain stimulation), IPG (implantable pulse generator), longevity, non-invasive, wireless charging

## Abstract

Deep brain stimulation (DBS) represents an important treatment modality for movement disorders and other circuitopathies. Despite their miniaturization and increasing sophistication, DBS systems share a common set of components of which the implantable pulse generator (IPG) is the core power supply and programmable element. Here we provide an overview of key hardware and software specifications of commercially available IPG systems such as rechargeability, MRI compatibility, electrode configuration, pulse delivery, IPG case architecture, and local field potential sensing. We present evidence-based approaches to mitigate hardware complications, of which infection represents the most important factor. Strategies correlating positively with decreased complications include antibiotic impregnation and co-administration and other surgical considerations during IPG implantation such as the use of tack-up sutures and smaller profile devices.Strategies aimed at maximizing battery longevity include patient-related elements such as reliability of IPG recharging or consistency of nightly device shutoff, and device-specific such as parameter delivery, choice of lead configuration, implantation location, and careful selection of electrode materials to minimize impedance mismatch. Finally, experimental DBS systems such as ultrasound, magnetoelectric nanoparticles, and near-infrared that use extracorporeal powered neuromodulation strategies are described as potential future directions for minimally invasive treatment.

## Introduction

Since its inception, deep brain stimulation (DBS) has revolutionized the management of a broad range of neurological and psychiatric diseases, from movement disorders to epilepsy and obsessive-compulsive disorder. Promising clinical trials have shown preliminary safety and efficacy of DBS as a treatment for disabling symptoms of Alzheimer’s disease, depression, and many other conditions (Lozano and Lipsman, 2013; Lozano et al., 2017). The unique ability of electrical modulation of the brain circuits with spatial and temporal accuracy enabled a completely new treatment paradigm complementing pharmacological approaches and lesioning procedures, which lack spatial and temporal control, respectively.

The success of DBS therapy depends not only on patient and target selection but also on the hardware used to generate and deliver the current. The implantable pulse generator (IPG) represents a key part of DBS systems and is the only component that requires programming, recharging, and potential replacement. The goal of the present work is to review the clinical challenges associated with current IPG design, IPG-related complications, and highlight future strategies to improve IPG longevity and practicality. The future potential of extracorporeal powered DBS systems is also briefly explored.

## Current IPG Design and Related Clinical Challenges

**Figure 1:**
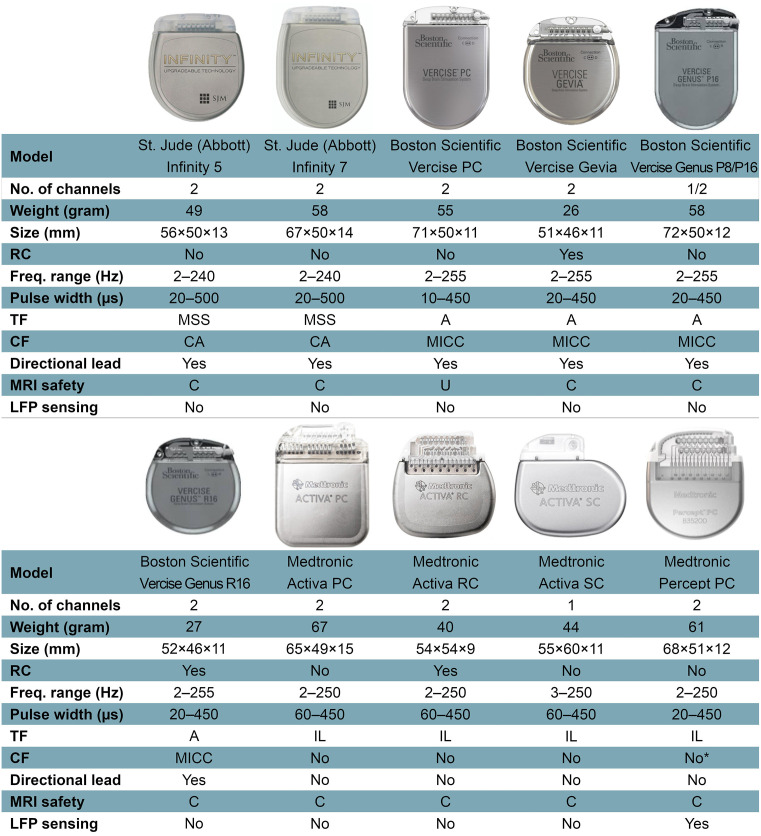
Features of current commercially available internal pulse generators. Abbreviations: A, areas; C, conditional; CA, coactivation; CF, current fractionation; Freq., frequency; Hz, Hertz; IL, interleaving; LFP, local field potential; MICC, multiple independent current control; MRI, magnetic resonance imaging; MSS, multi-stim set; PC, primary cell; RC, rechargeable cell; SC, single cell; TF, temporal fractionation; U, unsafe. *Percept PC can provide independent current control across 16 electrode contacts, but this function is not yet available on physician’s programmer as of March 2021. Not all features or devices are or will be available for a given region and are subject to local regulatory approvals.

The IPG is the active component of current DBS systems. It contains a battery and a power module, a CPU and program memory, as well as a microprocessor managing all the device’s functions, including activation, deactivation, pulsing parameters, internal diagnostics, and communication with external devices. Some IPGs also include recharging capabilities, integrated accelerometers, local field potential (LFP) sensing, onboard signal processing, and analysis capabilities. The technical features of current commercially available IPGs are portrayed in Figure 1.

### Clinical Challenges With IPGs

#### Inadequate Longevity and Frequent Replacement Surgeries

Battery longevity describes the period, during which a single IPG will successfully deliver the desired current before surgical replacement. IPG replacement is estimated to account for about 9% of the total cost of DBS therapy in short–term studies but proportionally increases over the lifetime of the patient (Dang et al., 2019). Each IPG replacement surgery is an additional economic, social, and psychological burden for the patient and workload/stress for the clinician. Moreover, subsequent surgeries bring additional complication risks to the patients and their DBS systems (Thrane et al., 2014; Fytagoridis et al., 2016; Frizon et al., 2017; Helmers et al., 2018). Thus, maximizing battery longevity should be a priority in the field.

Battery longevity depends on stimulation parameters, hardware, and patient factors (Bin-Mahfoodh et al., 2003; Fisher et al., 2018; Sette et al., 2019). Patient factors, such as reliability of IPG recharging or consistency of nightly device shutoff, if appropriate and tolerated (e.g., essential tremor, pain), may affect battery longevity. Hardware factors include the battery type (primary cell vs. rechargeable), chemistry and capacity, as well as energy consumption of the idle device. The impedance of the system, which is also a vital factor that affects battery longevity, can be both hardware and tissue-related factor (Butson et al., 2006). Stimulation parameters are the key determinant of the total power, which is strongly correlated with battery life (Fakhar et al., 2013). It is the only battery longevity affecting factor that can be modified by the clinician after DBS implantation. As DBS programming is extensively discussed elsewhere (Ramirez-Zamora et al., 2015; Picillo et al., 2016a, b), we will only briefly mention some of the relatively new, longevity-affecting stimulation techniques that may help to understand the features of IPGs more easily. *Constant-current stimulation (CCS)* is the consistent delivery of electricity to target by compensation for variations in impedance over time. Dynamic voltage changes during CCS have been associated with a greater battery consumption compared to constant-voltage stimulation in the short–term, although this difference disappears over long-term follow-up (Lettieri et al., 2015; Rezaei Haddad et al., 2017). The effect of battery longevity of *Bipolar stimulation*, in which one contact serves as the cathode while another serves as the anode, is disputable. While an earlier study appears to demonstrate an increase in longevity with bipolar stimulation compared to cathodic monopolar stimulation with Medtronic Soletra IPGs (Almeida et al., 2016), we demonstrated a higher battery consumption index with bipolar stimulation with Boston Scientific IPGs in one of our recent studies (Soh et al., 2019). This discrepancy might be due to differences between the devices or battery consumption index calculation methods, as well as the use of different amplitude values for bipolar stimulation between the studies (Soh et al., 2019). *Directional current steering* technologies have a complex impact on battery longevity, which will be discussed in detail in the Future Strategies section (see “Directional DBS” under Future Strategies). *Temporal fractionation* [“interleaving stimulation” as introduced by Medtronic and “Multi-stim Set (MSS)” by St. Jude/Abbott] uses two separate sets of stimulation parameters in an alternating fashion to shape the volume of tissue activation (VTA) delivered through a single DBS electrode. The alternating stimulation programs must share a common frequency but may have different amplitudes, polarities, and pulse widths. It may reduce battery longevity due to the increased pulses required (Ramirez-Zamora et al., 2015). *Vertical current fractionation* involves multiple independent current sources, which apply constant current through each contact of the DBS electrode. Boston Scientific IPGs use *multiple independent current control (MICC)* to control the flow of current through each contact, individually. The safety and efficacy of MICC for STN DBS in PD patients were demonstrated by a double-blind, randomized controlled INTREPID trial (Vitek et al., 2020), however, there are implications for IPG depletion depending on the settings utilized. Abbott IPGs use a less versatile method termed *Coactivation*, which allows for multiple contacts to be stimulated as if they were a single electrode, i.e., no independent control at each contact is possible. There are a limited number of articles that compare the current steering techniques between each other, as well as with conventional monopolar stimulation in regards to energy consumption. A computational modeling study showed that MSS may draw more or less battery current than MICC, while coactivation consistently draws less battery current than both MICC and MSS (Zhang S. et al., 2020). A human study in a Parkinsonian DBS cohort demonstrated that MICC significantly lowered total electrical energy delivered (TEED) compared to monopolar stimulation while similarly improving the functional ambulatory performance (Hui et al., 2020).

Disease-specific longevity is a tremendously important factor that necessitates the discussion with the patients prior to DBS surgery. It is well accepted that IPG longevity varies between conditions due to the variable energy requirements necessary to achieve therapeutic benefit. For example, dystonia and depression often require higher energy settings compared with ET and PD, which depletes the IPG faster (Rawal et al., 2014). This has considerable implications for the patient as well as the treating physician and patients should be made aware of what this may mean for their treatment in regards to the frequency of battery replacements.

Suboptimal DBS lead placement is another factor that may have an effect on battery longevity. Theoretically, a lead implanted away from the target zone, where stimulation produces above-mean clinical improvement (“hotspot”; Elias et al., 2021) necessitates a larger VTA to engage with the hotspot which in turn results in more energy consumption. This concept must be balanced with the possibility that sub-optimally positioned leads, depending on the vector of deviation from intended and/or the clinical hotspot, may actually limit the maximum voltage/amplitude due to the induction of off-target side-effects. Illustrative of this, Anheim et al. (2008) demonstrated in their prospective study that stimulation-induced adverse effects occur at lower voltage thresholds for the misplaced leads (mean 2.6 V) compared to the optimally placed leads (mean 4.4 V). The lower threshold for adverse effects prevents the use of sufficient energy to achieve an optimum clinical outcome in real-life circumstances, which prolongs battery longevity. Thus, a balance between the energy required for hotspot stimulation and optimal placement of leads with sufficient thresholds for off-target side-effects is of critical importance. Techniques for targeting accuracy using microelectrode recording, impedance monitoring and/or micro/macrostimulation have been long utilized in DBS surgery and were discussed previously in great detail (Hariz, 2002). The insertional effect, which transiently alters parenchymal impedance, may further complicate interpretation of the therapeutic stimulation window intra-operatively but experienced teams can incorporate these data in decision making for final lead placement intra-operatively. Finally, all electrodes should be verified by an imaging modality as an added confirmatory step. In addition to traditional verification techniques utilizing frame-based systems and fluoroscopy, verification of leads can also be achieved with intra-operative CT and/or 3D fluoroscopy. More recently, the use of intraoperative MRI for targeting and electrode guidance has increased in popularity and is routinely used as part of some surgical workflows (Hwang et al., 2021).

Recent battery longevity studies have shown that the newer generation IPGs have decreased battery lifespans compared to their predecessors. For example, the battery lifespan of the Activa PC is 3–4.6 years, compared to the Kinetra, which is 4.3–6.5 years (Fisher et al., 2018; Kiss and Hariz, 2019; Sette et al., 2019; Paff et al., 2020). On the other hand, the lifespan of rechargeable IPGs is estimated to range from 15 (Medtronic) to 25 (Boston Scientific) years, which has yet to be confirmed (Paff et al., 2020). Strategies for deciding between rechargeable and non-rechargeable IPGs have been discussed in detail elsewhere (Okun, 2019; Paff et al., 2020). Another consideration in addition to selecting a rechargeable IPG in patients, who initially were treated with a unilateral system but later needed a contralateral side treatment, is whether conversion to a dual-channel IPG should be considered. The mean longevity of a single channel Activa SC (37 months) is comparable to the longevity of the dual-channel Activa PC (Park et al., 2018). Thus, implanting two Activa SC IPGs may double the number of replacement surgeries required unless the IPGs are depleting simultaneously. At the same time, this must be weighed against the risk of compromising the first implanted unilateral system while tunneling the additional extension wire for the contralateral system. At our center, we often will discuss the pros and cons of both approaches with the patient and defer to patient preference, if there is equipoise between the two strategies.

#### Bulky Size of the IPGs and Skull Mounting

The size of currently available DBS IPGs necessitates their implantation on the chest wall, as opposed to the skull. The need for tunneling of extension wires to connect the DBS wires in the frontal skull region to the IPG located in the pectoral region requires general anesthesia, increasing the complexity of the second-stage surgery. Additionally, the bulk of the IPG case may cause wound dehiscence, skin erosion, and cosmetic problems, particularly in thin patients. The smaller profile of rechargeable IPGs compared to non-rechargeable IPGs has reduced wound healing and cosmetic problems to some extent (Figure 1). Taking into account the thickness of the skull, which is 7–8 mm on average in the frontoparietal region and changes with age (Lillie et al., 2016), the need for even smaller profile IPGs is essential for skull-mounting. The Neuropace responsive neurostimulation (RNS) system (Neuropace, Inc., USA), which includes a skull mounted IPG with maximum dimensions of 60 × 27.5 × 7.5 mm, was approved by FDA in 2013 for epilepsy and has been under use and/or investigation for the treatment of various diseases including epilepsy, Tourette syndrome, binge eating disorder, major depression, posttraumatic stress disorder, and anxiety disorders (Nair et al., 2020; Jarosiewicz and Morrell, 2021). The Neuropace stimulator is placed within a ferrule, which is secured to a full-thickness craniectomy and can be connected to one or two leads (depth or strip), which may be used for stimulating and/or sensing (Jarosiewicz and Morrell, 2021). Neuropace can deliver current-controlled, charge-balanced biphasic pulses with customized stimulation frequency (1–333 Hz), current (0.5–12 mA), pulse width (40–1,000 μs), and stimulation burst duration (10–5,000 ms; Morrell and Halpern, 2016). The most recent, MRI-conditional, RNS-320 has an expected battery life of ~8.4 years under moderate stimulation settings, in which <5 min of stimulation per day is delivered (Jarosiewicz and Morrell, 2021).

Implantation of IPGs within the skull raises the possibility of new concerns and complications. The spread of infection to the skull, meninges, and brain parenchyma may be of more concern due to their proximity to the brain compared to conventional IPGs. However, a 9-year prospective safety report of RNS systems demonstrated that there were no instances of meningitis or cerebritis among a total of 35 infections over the cumulative 1,895 patient-implantation years, and only one case of osteomyelitis has been reported (Nair et al., 2020; Razavi et al., 2020). Other possible concerns are potential imaging artifacts caused by the device during neuro-imaging and utilization of these systems in younger patients with growing skulls. Additional considerations are increased difficulty of revision surgery due to bony remodeling and the increased potential for brain lead fracture if there is no strain relief between the IPG and electrodes. In addition to the Neuropace system, another skull-mounted system-Picostim (Bioinduction, Bristol, UK)—is currently under trial (SPARKS trial) for CE approval in Parkinson’s disease patients (ClinicalTrials.gov NCT03837314). Developing skull-mounted systems for routine indications for DBS is a priority in the field as it has advantages from both clinical (e.g., surgery can be completed in a single stage without general anesthesia) and patient perspective (e.g., cosmesis). Care will have to be taken in the design and deployment of this approach, especially if rechargeable systems are considered.

#### Challenges With Recharging of the IPGs

Rechargeable IPGs have successfully enriched the armamentarium of the DBS clinicians with their increased longevity and smaller size. Despite these clear advantages, there are some drawbacks that limit their utilization. While recharging is generally considered easy and convenient, these devices might not be suitable for patients with advanced age and cognitive problems that might prevent them from being able to consistently recharge their devices (Jakobs et al., 2019). Current rechargeable IPGs require a minimal distance between the charging pad and the IPG during the charging session. Some patients find pairing the charging pad and IPG difficult, feel “tethered” during charging, or find it cumbersome to track the charge level of the device (Mitchell et al., 2019). The charge burden is variable among patients and depends on the diagnosis, IPG model, and stimulation parameters. The reported average time of charging is 185.8 (range: 25–830) minutes divided over a mean of 4.5 (range 0.5–14) charging sessions per week, which is perceived as reasonable to most patients (Mitchell et al., 2019). From a surgical point of view, the necessity of superficial IPG implantation (1–1.5 cm beneath the skin surface) may predispose some thin patients to skin erosions.

#### MRI Compatibility

Around 70% of patients will need an MRI within 10 years of DBS implantation due to comorbidities or device complications (Falowski et al., 2016). MRI-related injuries in the early 2000s in DBS patients led to a considerable number of MRI safety studies being conducted and establishment of MRI guidelines by hardware vendors (Boutet et al., 2020). Fortunately, MRI compatibility of newer devices is improving with almost all currently available IPGs being full-body 1.5 Tesla MRI-conditional (Figure 1). Additionally, the new Medtronic IPG, Percept PC, has been tested in 3.0-T MRI environments and found to be MRI-conditional when eligibility criteria are fulfilled. Nevertheless, patients with other IPGs may also be scanned with a 3.0-T MRI, currently off-label but with promising phantom study data which with further characterization from other centers will hopefully enable broadening indications (Boutet et al., 2019). On the other hand, patients implanted with older generation devices may face delays or contraindications to neuroimaging.

#### Limited Number of Lead Channels

Some clinically complex movement disorder patients may need multitarget DBS and more than two leads concurrently (Parker et al., 2020). While there are spinal cord stimulation IPGs with four channels, commercially available DBS IPGs have a maximum of only two channels, which results in the implantation of at least two IPGs for this rare patient subpopulation.

#### Local Field Potential (LFP) Sensing Quality

The use of LFP sensing is important in adaptive therapeutic stimulation, as well as in acquiring basic neuroscientific research data by neural recording over time in out-of-clinic environments. In addition to the aforementioned NeuroPace device, Medtronic has released several IPGs with sensing abilities. Initially, as research devices, the first-generation IPG of its kind (Medtronic Activa PC+S) had limitations in signal sensing quality, management of the stimulation and other artifacts, and long–term data recording (Swann et al., 2018a). Even though the second-generation IPG (Medtronic Summit RC+S) provided a substantial improvement over the precedent (Stanslaski et al., 2018), it was not commercialized, whereas Medtronic Percept PC has been commercially available since 2020. The new device can capture LFP signals and allow clinicians to review these signals with respect to custom patient-reported events (i.e., ON or OFF medication state, dyskinesia, tremor, took medication, etc.). The survey mode allows displaying—LFP magnitude (microvolts peak) vs. a frequency band (0–100 Hz)—for all possible contact pairs while the stimulation is off. In a streaming mode, real-time visualization of the stimulation amplitude and the LFP power of a pre-selected frequency band (selected frequency ±2.5 Hz) from a single pre-defined contact pair is possible. While capturing the LFP power in the selected frequency band, the clinician can turn on the stimulation and see the real-time changes in the LFP power while changing the stimulation amplitude. This mode has an online sampling frequency of 2 Hz, but the raw data is sampled at 250 Hz which can be analyzed offline at a later timepoint. There are two low-pass filters at 100 Hz and two high-pass at 1 Hz and, 1 or 10 Hz as defined by the clinician. Some of the limitations of this new IPG are the necessity of two sensing contacts on lead, inability to stimulate on sensing contacts, monopolar/double monopolar stimulation only through contact(s) between sensing contacts, no interleaving, and increased noise with stimulation amplitudes over 5 mA. In addition, the stimulation rates must be at least 10 Hz greater than the selected LFP band of interest (Thenaisie et al., 2021).

#### In-Person vs. Remote DBS Programming

A secure, web-based, remote, wireless programming system for DBS has been implemented in China since 2014 (Zhang J. et al., 2020). This system is currently available for the IPGs manufactured by PINS Medical Co., Ltd. (Beijing, China) and SceneRay Co., Ltd. (Suzhou, China) and allows clinicians to adjust DBS settings of patients remotely without the necessity of coming to hospital or clinic (Paff et al., 2020). More recently, Abbott announced the launch of its FDA-approved NeuroSphere Virtual Clinic that allows remote programming. Such a feature is paramount with many patients coming from a great distance to specialized centers and in-person programming has been further challenged by the COVID-19 pandemic (Fasano et al., 2020).

## IPG-Related Complications and Avoidance Strategies

IPGs can be associated with a number of complications, which constitute a major priority for the multidisciplinary team to anticipate, prevent, and manage. The main IPG-related complications include infection, flipping, skin erosion, malposition, and malfunction (Table 1; Fenoy and Simpson, 2014; Jitkritsadakul et al., 2017). These complications not only cause interruption of therapy but inflict a great economic cost. The cost of a single DBS system removal or revision is approximately US$ 12k, while the average reimplantation cost of a DBS system can reach up to US$ 41k depending on the health system and IPG model used (Chen et al., 2017; Wetzelaer et al., 2018). As the overall cost of health care is rising in many countries, efforts to reduce excess costs related to surgical site infections and other complications are paramount. Herein, we discuss the most common early and delayed IPG-related complications while highlighting strategies for prevention and management.

**Table 1:** IPG-related complications and avoidance strategies.

IPG-related complications	Potential avoidance strategies
Infection	Prophylactic perioperative antibiotics Vancomycin powder. Antibiotic envelopes Decreasing the number of replacement surgeries by using long-lasting IPGs (i.e., rechargeable IPGs). Prevention of CSF leak into the pocket.
Subcutaneous seroma/hematoma in the vicinity of the IPG	Avoidance of over-sized IPG pockets Proper hemostasis during surgery. Prevention of CSF leak into the pocket.
Skin erosion	Deeper implantation of the IPG. Proper fixation to decrease motion. Antibiotic envelopes
Wound dehiscence / Exuberant scarring of the wound	Avoidance of small-sized IPG pockets Decrement in the size of IPG.
Uncomfortable feeling around IPG	Subpectoral implantation (Son et al., 2012)
Flipping (Twiddler’s syndrome)	Subfascial/submuscular placement of the IPG Two-point anchorage with non-absorbable suture/stitching the pocket to reduce its size Antibiotic envelopes/polyester pouches
Malposition / Migration	Proper fixation Changing to a lower profile IPG Antibiotic envelopes
Malfunction	-
Ineffective recharging / Shielded Battery Syndrome	Implantation of IPG no more than 1.5 cm beneath the skin. Fixation of the adaptor beneath the IPG.

### Early Complications

Theoretically, several different IPG-related complications can be encountered at any time after the implantation but some are more prone to happen earlier in the first 3–6 months, while some more often occur in a delayed fashion. Among the IPG-related early complications, the most serious is an *infection*, which, in severe cases, may necessitate the removal of all DBS hardware (Voges et al., 2006; Fenoy and Simpson, 2014). Other IPG-related early complications include the development of *subcutaneous seromas or hematomas* in the vicinity of the IPG, *skin erosion, wound dehiscence, IPG flipping, ineffective recharging, malposition, uncomfortable feeling around IPG*, and *malfunction* primarily due to faulty production (Voges et al., 2006; Fenoy and Simpson, 2014; Benam et al., 2019).

DBS hardware infection has a reported incidence of up to 15% of cases (Joint et al., 2002; Oh et al., 2002; Voges et al., 2006; Sillay et al., 2008; Fenoy and Simpson, 2014), with most occurring within 6 months of surgery (Sillay et al., 2008; Fenoy and Simpson, 2012; Frizon et al., 2017). The IPG-originated infection rate is reported as 2% after the primary implantation and ranging from 0.7% to 6% for IPG replacement surgeries (Thrane et al., 2014; Fytagoridis et al., 2016; Frizon et al., 2017; Helmers et al., 2018). Most case series suggest the rate of infection is increasing with the number of previous replacement procedures (Thrane et al., 2014; Fytagoridis et al., 2016; Helmers et al., 2018), while Frizon et al. (2017) demonstrated the opposite, with infection rates of 0.4% for the 1st, 1.8% for the 2nd and 0% for the 3rd and subsequent replacement surgeries. IPG infections typically present with erythema, swelling, and purulent discharge from the pulse generator pocket incision. The most commonly identified infectious agents are *S. epidermidis* and *S. aureus*, with the latter being the most difficult to treat without hardware removal (Sillay et al., 2008; Fenoy and Simpson, 2012; Frizon et al., 2017; Helmers et al., 2018). Avoidance of infection must be one of the highest priorities at the time of surgery. Some evidence suggests spreading vancomycin powder throughout the IPG pocket during insertion may reduce infection rates (Rasouli and Kopell, 2016; Abode-Iyamah et al., 2018). Vancomycin powder is inexpensive and widely available. Additionally, the administration of perioperative antibiotics should follow local protocols and typically does not exceed 24 h.

For the past decade, antibiotic envelopes have been implemented for cardiac implantable electronic devices (CIED) to prevent infection. As an example of antibiotic envelopes, the TyRx (Medtronic, Dublin, Ireland), which contains rifampin and minocycline, prevents hardware infections by eluting these antimicrobial agents in the local tissues for more than 7 days following the procedure. Antibiotic envelopes may also prevent IPG migration, erosion, or Twiddler syndrome as a result of its porous mesh structure that triggers dense fibrous connective tissue ingrowth (Osoro et al., 2018). Several reports related to the field of cardiac surgery have demonstrated that antibiotic envelopes are both effective and cost-efficient (Tarakji et al., 2019; Mittal et al., 2020; Pranata et al., 2020). A large, multicenter, randomized trial including 6,983 patients (Tarakji et al., 2019; Mittal et al., 2020) reported a 40% reduction in major CIED infections and a 61% reduction in pocket infections within 12 months of placement. While antibiotic envelopes have yet to be studied for infection prevention in DBS patients, it seems reasonable to apply these findings to DBS IPG insertion considering the similar size and implant location especially in the case of implanting an IPG in a higher risk patient (e.g., diabetic, immunosuppressed, etc.).

When an IPG infection does occur, antibiotic therapy should be initiated immediately in an attempt to save the DBS system and prevent more rare and severe complications such as cerebritis and brain abscess. Algorithms for managing DBS hardware infections vary among institutions. Depending on the severity of the infection, some centers may initiate a trial of antibiotic therapy while others will promptly remove the IPG and/or other portions of the hardware in addition to treatment with intravenous antibiotics between 4–8 weeks. Once the infection is cleared, IPGs can be safely re-implanted after 2–3 months (Lyons et al., 2004; Temel et al., 2004; Sillay et al., 2008; Boviatsis et al., 2010; Fenoy and Simpson, 2012). If there is a high risk of withdrawal syndrome, IPG and extension cables can be removed and a contralateral side IPG with new extensions can be implanted in the same operative session under appropriate antibiotics (Helmers et al., 2021). For patients with high stimulation settings necessitating frequent battery changes, switching to a long-lasting IPG [i.e., rechargeable Activa RC or Vercise Gevia are estimated to have life-spans of >15 years (Thrane et al., 2014; Fytagoridis et al., 2016; Helmers et al., 2018)] should be considered as a means of reducing the risk of infection from repeated surgical procedures, as well as healthcare costs (Hitti et al., 2018).

*Ineffective recharging* of rechargeable IPGs may occur when the IPG is implanted too deep beneath the skin and/or at a suboptimal angle to allow effective communication between the IPG and recharging device. Per manufacturer recommendations, rechargeable IPGs should be implanted approximately 1.5 cm beneath the skin. In the case that an adaptor has been used to connect an older generation DBS lead system to a new-generation rechargeable IPG, it is possible for the adaptor and wires to migrate between the IPG and the skin, impeding the recharging process. This situation has been termed “*shielded battery syndrome*.” In the case of shielded battery syndrome, relocation of the wires and adaptor is necessary (Chelvarajah et al., 2012).

### Delayed Complications

Delayed complications of IPGs arise mostly due to suboptimal fixation or placement and device wear and tear. The incidence of *IPG malfunction* is reported in the literature as 0.1% to 13.8% (Lyons et al., 2004; Doshi, 2011; Umemura et al., 2011; Fenoy and Simpson, 2014). Device malfunction should be suspected when the IPG does not respond during interrogation, or when there is an unexplained decline in clinical benefit. Hardware damage, such as fractured DBS leads and extension wires, should be ruled out with X-ray and impedance testing. If IPG malfunction is suspected and other causes of system malfunction have been excluded, exchange of the IPG is unavoidable (Lyons et al., 2004; Blomstedt and Hariz, 2005).

Infection can also be seen as a late complication. Frizon et al. (2017) demonstrated that 20% of all IPG-originated infections occur after 6 months; however, in their series they could not identify a variable associated with a significant increase in the risk of infection, such as steroids, anticoagulant, and aspirin use; body mass index; hypertension; diabetes mellitus; and coronary artery disease. Although these variables may theoretically increase the infection rates, this has not been borne out in the DBS case series that present long-term complication rates (Baizabal Carvallo et al., 2012; Frizon et al., 2017).

Other late complications of IPGs may arise from *suboptimal positioning*. Over time, poor positioning of the IPG can lead to discomfort and/or poor cosmesis. In their 728-patient DBS cohort, Fenoy and Simpson reported only four patients (0.5%), who required a repositioning surgery due to a flipped, uncomfortable or malpositioned IPG (Fenoy and Simpson, 2014). There are reports suggesting subpectoral IPG implantation over subcutaneous implantation to achieve a more favorable cosmetic outcome, as well as less patient discomfort (Son et al., 2012; White-Dzuro et al., 2017). *Exuberant scarring of the IPG wound* may cause both poor cosmetic results and bowstringing (wire tethering), which is a considerable cause of pain-related discomfort and limitation of neck movements in DBS patients (Miller and Gross, 2009). *Migration of the IPG* can occur, especially with older IPG models. In such cases, revision of the subcutaneous pocket or relocation is warranted (Blomstedt and Hariz, 2005; Messina et al., 2014). Changing to a lower profile rechargeable IPG can help in such situations. *Skin erosion* *over the IPG* is another challenge, especially if the skin of the patient is very thin, which is a common issue with dystonic and anorexic patients (Frizon et al., 2017).

Another delayed complication involves twisting of the extension wires as the IPG flips over within the subcutaneous pocket. Although different types of flipping syndromes are described in CIED literature, only *Twiddler’s syndrome* (IPG rotation around its vertical axis) has been described in DBS patients, which typically presents with DBS system malfunction. Its prevalence was reported as 1.3–1.4% of all DBS implanted patients in two different case series (Burdick et al., 2010; Sobstyl et al., 2017). A plain X-ray will show twisting of the extension wires often accompanied by migration or fracture of the extension wires or leads (Sobstyl et al., 2017). Twiddler’s syndrome is mitigated with subfascial/submuscular placement of the IPG with two points of anchorage with non-absorbable suture and stitching the pocket to reduce its size (Sobstyl et al., 2017), as well as antibiotic envelopes or polyester pouches may be useful by increasing fibrous tissue formation that may limit IPG movement (Osoro et al., 2018). Some of these complications are illustrated in the photographic and radiographic form in the review by Morishita et al. (2010).

## Future Strategies to Improve IPG Longevity and Practicality

Recent innovations that have the potential to improve IPG longevity and/or practicality include novel stimulation patterns, material properties of the DBS system, skull-mounted generators, as well as enhanced wireless power transfer techniques.

### Improving IPG Longevity by Alternative Stimulating Patterns

#### Directional DBS

Directional DBS (dDBS) refers to DBS with segmented leads that allow for shaping the electrical field perpendicular to the lead towards a specific brain region. Rebelo et al. (2018) provided some of the first evidence that the dDBS can consume less energy than conventional DBS (cDBS). They reported a 31% reduction in therapeutic current strength (TCS) and an overall 6% decrease in TEED compared to that estimated for all leads programmed as the best omnidirectional alternative. Similarly, in the early results of the Abbott-sponsored PROGRESS trial, dDBS achieved a similar clinical benefit compared to cDBS at a significantly lower (39%) TCS, which may have a considerable effect on energy consumption (Ramirez-Zamora et al., 2020) (www.ClinicalTrials.gov NCT02989610). Programing of directional leads is slightly different from the programming of conventional leads, as the density of charge is higher given the small surface of these segmented leads. The maximally allowed amplitude is 3.4 mA per contact based on the recommended threshold of tissue damage on the charge density of 30 mC/cm^2^ (Pollo et al., 2014). Understanding the nuances of dDBS programming is paramount to maximizing the potential energy savings of such systems.

#### Cycling DBS

ON/OFF cycling is a frequently used parameter, particularly for the anterior nucleus of thalamus stimulation in epilepsy patients (Fisher et al., 2010). It is a potential approach to reduce energy delivery; however, acute stimulation studies showed a decreased treatment effect with cycling DBS compared to conventional DBS in ET (Swan et al., 2016), PD (Montgomery, 2005), and epilepsy (Molnar et al., 2006) patients. To demonstrate the efficacy of cycling DBS in ET patients, a prospective, randomized, double blind clinical trial has been designed and it is currently recruiting patients (www.ClinicalTrials.gov NCT04260971). Utilization of *Theta Burst DBS*, cyclic stimulation for 100 ms followed by a pause of 100 ms (Horn et al., 2020) or 200 ms (Sáenz-Farret et al., 2021) with a pulse width of 60 μs and a frequency of 50 Hz, may be beneficial for refractory axial symptoms of PD patients. Further research including battery consumption is needed in this field.

#### Ramped-Frequency DBS

Swan et al. (2020) recently evaluated a novel stimulation pattern termed ramped-frequency stimulation (RFS) in ET patients. These RFS patterns consisted of a harmonic progression of 15 instantaneous pulse frequencies that decreased from 130 Hz to 50 Hz, 130 Hz to 60 Hz, or 235 Hz to 90 Hz. These patterns were compared with constant frequency stimulations (CFS) that correspond to the mean pulse rates of the respective RFS patterns. Significant tremor suppression relative to “off” stimulation was shown with three different stimulation parameters: (i) 130 Hz CFS (greatest symptom relief), (ii) 82 Hz CFS, and (iii) 130–60 Hz RFS. There were no significant differences in tremor suppression between any RFS trains and their respective frequency-matched CFS trains. Thus, they suggested that tremor-related thalamic burst activity might result from burst-driver input, rather than from an intrinsic rebound mechanism. RFS may exacerbate thalamic burst firing by introducing consecutive pauses of increasing duration to the stimulation pattern. The balance between the energy conservation by the reduction of the average frequency of stimulation with RFS and the energy expenditure to drive this pattern is not known and warrants further investigation.

#### Square Biphasic Pulse DBS

The cDBS waveform consists of a rectangular biphasic pulse, with an active, high-amplitude and short-duration phase, followed by a passive, low-amplitude, and charge-balancing phase. Using square biphasic (sqBIP) pulses (with active rather than passive charge-balancing phase) is a novel method and shows similar, or even greater therapeutic benefit over cDBS in the treatment of PD, ET, and dystonia patients (Akbar et al., 2016; Almeida et al., 2017; De Jesus et al., 2019). However, the battery consumption was found significantly higher in sqBIP DBS than cDBS (Akbar et al., 2016), thus the utility of sqDBS with current non-rechargeable IPG configurations may be of limited value.

#### Replacing High-Frequency Stimulation With Low-Frequency

Low-frequency stimulation (LFS, <100 Hz) in PD has several advantages and drawbacks compared to conventional high-frequency stimulation (HFS, >100 Hz; Di Biase and Fasano, 2016; Su et al., 2018). LFS may be superior to HFS in akinesia, gait, and freezing of gait sub-scores, whereas HFS may induce better responses for tremor. LFS is associated with a decrease in the total electrical energy delivery and may help extend battery longevity. The mechanism of action of LFS may be different from that of HFS (i.e., maximum effectiveness achievement in ventral STN, or its possible effects on PPN activity; Su et al., 2018), which necessitates further evaluation before routine clinical application.

In 2017, Brocker et al. (2017) used a genetic algorithm (GA), which is an optimization technique based on principles from biological evolution, to design an optimized temporal pattern of stimulation. They coupled GA with a model of the basal ganglia in the design of an optimized stimulation pattern. The authors found out that the GA DBS (average frequency of 45 Hz) performance was equivalent to high-frequency (185 Hz) DBS in the bradykinesia-related finger-tapping task. The predicted changes in UPDRS motor sub-scores produced by stimulation with the GA pattern were equivalent to those produced by 185 Hz. However, the suppression of Parkinsonian tremor by GA DBS was somewhat lower than by HFS, which is in line with the abovementioned studies comparing LFS with HFS.

#### Variable Frequency Stimulation (VFS)

This is a novel DBS paradigm consisting of delivering both HFS and LFS interleaved in varying patterns using the PINS Medical IPGs (Jia et al., 2018). In a four patient pilot study, VFS was found superior to conventional HFS in the treatment of appendicular, as well as axial symptoms and freezing of gait (Jia et al., 2018). The effect on battery conservation is unknown and yet to be investigated.

#### Adaptive DBS

Adaptive DBS (aDBS; closed-loop or responsive DBS) is a technique in which the delivery of the stimulation is modulated by the real-time sensing data *via* a feedback mechanism. aDBS can be amplitude-responsive, which refers to using the amplitude of signals to estimate the degree of circuit dysfunction, i.e., level of beta (13–30 Hz) LFP activity in STN (Kühn et al., 2008), or phase (timing) responsive, where pulses of stimulation are timed to a particular phase as in the treatment of tremor (Meidahl et al., 2017). The goal of this type of stimulation is to widen the therapeutic window by optimizing the delivery of the stimulation to correct the degree of circuit dysfunction. Transitioning from continuous stimulation to the responsive stimulation of aDBS is also expected to decrease the amount of energy consumption. Furthermore, several human clinical trials (Little et al., 2013, 2016; Rosa et al., 2017; Swann et al., 2018b; Velisar et al., 2019; Opri et al., 2020; He et al., 2021) have assessed the average energy saving associated with aDBS compared to continuous DBS in a similar time period and showed a range of energy-saving percentage of 38–73%. The characteristics and energy consumption percentages of these trials are given in Table 2.

**Table 2:** Clinical trials of adaptive DBS with stated energy consumption.

Author, Journal, Year	Disease, Patient #, Target	Biomarker	Study protocol	Clinical effect	Mean Total Electrical Energy Delivered (TEED) during stimulation period	Average energy saving**
Little et al. (2013)	PD (8 patients), unilateral STN	LFP beta activity (if exceeds threshold, voltage increases)	DBS OFF, aDBS, cDBS and random DBS comparison *via* externalized extensions up to 7 days after lead implantation	Motor scores during aDBS improved better than cDBS by 29% (unblinded) and 27% (blinded)	aDBS (132 +/− 21 uW) cDBS*(270 +/− 37 uW) *p< 0.0001	51%
Little et al. (2016)	PD (4 patients), bilateral STN	LFP beta activity (if exceeds threshold, voltage increases)	DBS OFF and aDBS comparison *via* externalized extensions 2–6 days after lead implantation, L-dopa ON/OFF.	Motor scores are 43% better with aDBS than DBS OFF.	aDBS (223 + /− 31 uW) cDBS (estimated)(491 +/− 44 uW)	55%
Rosa et al. (2017)	PD (10 patients), unilateral STN	LFP beta activity (if exceeds threshold, voltage increases)	aDBS and cDBS comparison *via* externalized extensions 5 and 6 days after lead implantation, L-dopa ON/OFF	The clinical scores were not significantly different between aDBS and cDBS. aDBS was more effective on dyskinesias.	aDBS (44.6 + /− 47.9 uW) cDBS*(158.7 + /− 69.7 uW) *p< 0.0005	73%
Swann et al. (2018b)	PD (2 patients), unilateral STN	Cortical gamma band activity (if exceeds threshold, voltage decreases)	aDBS and cDBS comparison. aDBS delivered by Activa PC+S *via* Nexus D3 (patient tethered) and E interfaces (patient free).	Similar bradykinesia and dyskinesia scores for cDBS, Nexus D3 and E (Pt 1). N/A for Pt 2.	N/A	38% (Nexus D3) 39–45% (Nexus E)
Velisar et al. (2019)	PD (13 patients), 20 STN leads	LFP beta activity (dual threshold)	DBS OFF, aDBS and cDBS comparison. aDBS delivered by Activa PC+S *via* Nexus D3 interface.	aDBS significantly improved bradykinesia and tremor over DBS OFF.	N/A	44%
Opri et al., 2020	ET (3 patients), unilateral M1 subdural leads	#x02014;VIM DBS lead	Movement onset by LFP of M1 and VIM (15/25 Hz) (EMG and inertial sensors used only for tremor evaluation, not as inputs)	DBS OFF, aDBS and cDBS comparison. aDBS delivered by Activa PC+S *via* Nexus D/E interface. Longitudinal follow-up for 6 months.	aDBS and cDBS improved the contralateral tremor scores by 47% and 52% compared with DBS OFF, respectively	N/A	57% (in clinic) 50% (at home)
He et al. (2021)	ET (6 bilateral, 2 unilateral patients), VIM-ZI	VIM LFP while the patient performed tremor provoking movements (Trained models)	DBS OFF, aDBS and cDBS comparison *via* externalized extensions 4 or 5 days after lead implantation.	aDBS and cDBS suppressed the tremor by 52% and 53% compared with DBS OFF, respectively.	N/A	61%

DBS, Deep Brain Stimulation (aDBS: adaptive, cDBS: continuous); EEG, Electroencephalography; EMG, Electromyography; ET, Essential Tremor; FoG: Freezing of Gait; GPi, Globus pallidus internus; M1, primary motor cortex; LFP, Local Field Potential; N/A, Not-applicable; PD, Parkinson’s Disease; PPN, Pedunculopontine nucleus; Pt, Patient; STN, Subthalamic Nucleus; VIM, Ventral intermediate; ZI, Zona incerta. *Statistical significance presents. **Calculated by formula (TEED-cDBS − TEED-aDBS)/TEED-cDBS.

#### Computational Models and Functional MRI Response Patterns for Optimization of DBS Programming

Apart from stimulation patterns, using a neuroanatomically based computer model for programming in PD patients provides comparable efficacy and less battery consumption over traditional, monopolar review-based programming, which has been demonstrated by the pilot GUIDE trial (Pourfar et al., 2015). A recent advance in the field of DBS programming is utilizing fMRI response patterns and machine learning algorithms to optimize DBS parameters. Our group demonstrated that DBS at optimal settings in PD patients produces a characteristic brain activation pattern on functional MRI with selective recruitment of motor circuits. This pattern can be used to predict optimal stimulation settings for individual patients and early identification of optimal settings may improve IPG longevity (Boutet et al., 2021).

### Improving IPG Longevity by Electrode Material Selection

The conventional microelectrodes are comprised of noble metals such as gold (Au), Platinum (Pt), and Iridium (Ir), which are highly corrosion resistant in biofluids, however, their performance is limited by the mechanical mismatch between the electrode and neural tissue, which can lead to scarring, high impedance, and low surface area which restricts their charge injection capacity (CIC) (the maximum deliverable charge per unit area) (Cogan, 2008). Alternative materials have been under investigation for years with the goal of increasing the electrochemical surface area and reducing impedances. A lower impedance is expected to result in lower power usage and longer battery life. Examples of alternative microelectrode materials include ceramics (e.g., titanium nitride and iridium oxide), conducting polymers, nanoporous Pt, Pt grass, carbon nanotube arrays, and laser pyrolyzed graphene (Won et al., 2020). Recently, Wang et al. (2019) demonstrated the performance of microelectrodes made from graphene fibers coated with Pt. These microelectrodes have an unrivaled CIC with the ability to record and detect neural activity with an outstandingly high signal-to-noise ratio (SNR) in an area as small as an individual neuron; thus, making them potentially interesting candidates for use in closed-loop systems.

### Improving IPG Practicality by Using Enhanced Wireless Power Transfer Techniques

Current commercially available rechargeable systems use *near-field short-range* *inductive coupling* wireless technology, which allows for power transfer across an exclusively short distance. The distance between the charging pad and the IPG battery can be increased by different wireless power systems such as: (1) *Magnetic resonant coupling systems* (Shin et al., 2017); which comprise resonant circuits that greatly increases coupling and power transfer between coils; (2) *Far-field RF transmission systems* (Park et al., 2015), which uses high-gain antennas or optical systems that reflect and refract electromagnetic radiation into beams and focus them on the receiver; and (3) *Ultrasonically powered* (Hinchet et al., 2019) *or Solar-powered* (Tokuda et al., 2018)* Wireless Battery Systems*. All the above-mentioned technologies may enable area wireless power coverage in the future. Patients can hang a transmitter coil in the walls of their living rooms that will wirelessly power and recharge their batteries while they are freely moving in the house. A commercially available prototype-Freedom-8A Wireless Spinal Cord Stimulator System (Stimwave, Pompano Beach, FL, USA)—is composed of a surgically implanted stimulator lead and a receiver that receives energy from a wearable transmitter and a battery. The transmitter and battery couple, which is called “Wearable Antenna Assembly”, is worn above the skin, couples the RF energy on the receiver located under the skin, and can be recharged externally (Bolash et al., 2019). A similar system is available with a baseball cap implanted transmitter for peripheral nerve stimulation (StimRelieve LLC, Miami Beach, FL, USA; Weiner et al., 2017).

## Technological Advances Towards Extracorporeal Powered Non- to Minimal-Invasive DBS Systems

**Figure 2:**
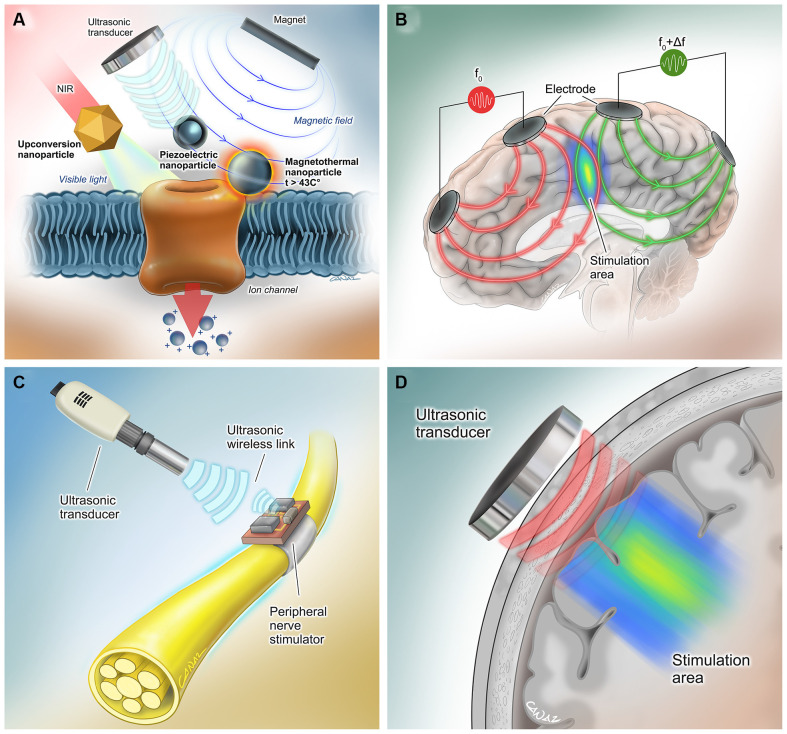
Extracorporeal powered non- to minimal-invasive DBS systems.** (A)** (Left) Transcranial near-infrared light can be converted to visible light by molecularly tailored upconversion nanoparticles for stimulation of genetically modified channelrhodopsin-expressing neurons (Chen et al., 2018). (Middle) Piezoelectric nanoparticles can activate neurons when they are powered using an external magnetic field (Kozielski et al., 2021) or ultrasound (Marino et al., 2015). No genetic modification is needed for this method. (Right) Another method involves activation of genetically modified heat-sensitive capsaicin receptors on neurons by heat- generating magnetic nanoparticles (Chen et al., 2015). **(B)** In the method by Grossman et al., multiple electric fields at frequencies too high to recruit neural firing, but which differ in frequency within the dynamic range of neural firing were delivered. The interference between the two applied fields served to cancel out the high-frequency activity but allowed the emergence of an oscillation corresponding to the difference in the two frequencies that allows electrical stimulation of the neurons in the interference region (Grossman et al., 2017). **(C)** Neurons can be activated by wireless, leadless, battery-free, and small (1.7 mm^3^ in volume) neural stimulators that are powered ultrasonically by hand-held external transceivers (Piech et al., 2020). **(D)** Transcranial ultrasound has the potential to be used as a neuromodulation tool even in the absence of a neurostimulator device (Fomenko et al., 2020). Reproduced with the permission of Dr. Gokhan Canaz (Cura Canaz Medical Arts).

With the unprecedented advancement in technology over the past few years, several approaches have been taken to activate neurons non- to minimal-invasively without requiring an internal power source. Some examples of such advances include ultrasonically powered systems, magnetically activated nanoparticles, temporally interfering electric fields, and near-infrared stimulation (Figure 2).

### Ultrasonically Powered Systems

Wireless, leadless, battery-free, and small (1.7 mm^3^ in volume), StimDust is a recently developed neural stimulator that is powered ultrasonically by a hand-held external transceiver. The system includes a piezoceramic transducer that acts as the antenna of the system, an energy-storage capacitor, and an integrated circuit, which can efficiently harvest ultrasonic power, decode downlink data for the stimulation parameters and generate current controlled stimulation pulses, even when embedded in porcine tissue at a depth of more than 5 cm. Safety monitoring and alignment are provided through an ultrasonic backscatter. *In vivo* efficiency was demonstrated by stimulating the sciatic nerve of rats, which resulted in neuronal activation (Piech et al., 2020). Ultrasound can also be exploited in combination with piezoelectric materials, such as barium titanate nanoparticles (BTNP), in order to generate direct-current output, induce Ca^2+^/Na^+^ influx, and elicit neural stimulation (Marino et al., 2015). In the future, a wearable ultrasound transceiver as a baseball cap may be utilized for neural stimulation in humans *via* this method, following minimally invasive implantation of such stimulator devices. Of note, ultrasound has the potential to be used as a neuromodulation tool, even in the absence of these millimeter-thick neurostimulator devices (Fomenko et al., 2020).

### Magnetoelectric and Magnetothermal Stimulation by Injectable Nanoparticles

This approach involves the use of magnetoelectric nanoparticles (MENPs) produced from magnetostrictive CoFe_2_O_4_ nanoparticles coated with piezoelectric BaTiO_3_ (Kozielski et al., 2021). *In vivo* studies have demonstrated that MENPs injected cells can be activated under remote non-resonant frequency magnetic stimulation, which is sufficient to cause neural activation to change animal behavior.

In another similar neural excitation technique, injected magnetic nanoparticles exploited thermal energy rather than generating electrical fields to activate genetically introduced heat-sensitive capsaicin receptor TRPV1 on neural cell membranes and elicit depolarization (Chen et al., 2015).

### Near-Infrared Stimulation *via* Upconversion Nanoparticles

The application of optogenetic methods in humans may be a revolutionary modality for neurostimulation. The first human optogenetic clinical trial has been ongoing for the treatment of retinitis pigmentosa patients (ClinicalTrials.gov NCT02556736). A demonstration of safety and feasibility in such a study may open the door for human research for deep brain stimulation *via* optogenetics. A promising study by Chen et al. demonstrated a novel DBS modality, in which extracranially applied tissue-penetrating near-infrared (NIR) light replaces the visible light source leads in conventional optogenetics. Molecularly tailored upconversion nanoparticles (UCNPs) were injected into deep brain tissues to convert transcranial NIR irradiation to visible light for activation of channelrhodopsin-expressing neurons (Chen et al., 2018). In the future, DBS may be performed using stereotactically injected viral vectors with UCNPs and wearable NIR light sources. However, this approach may be more difficult to deploy compared to the aforementioned alternatives, as it will require an exogenous expression of channelrhodopsins that, although feasible in preclinical models, still have significant hurdles to overcome for translation into human patients.

### Temporally Interfering Electric Fields

In 2017, Grossman et al. (2017) presented a method that enables noninvasive stimulation of deep brain structures by delivering multiple electric fields at frequencies too high to recruit neural firing, but which differ in frequency within the dynamic range of neural firing. The interference between the two applied fields served to cancel out the high-frequency activity but allowed the emergence of an oscillation corresponding to the difference in the two frequencies that allows electrical stimulation of the neurons in the interference region. The feasibility of this technique has been demonstrated in mice, whereas chronic application in human brains requires further investigation. However, this method has the potential to change conventional DBS methods and allow the externalization of power sources.

## Conclusion

At present, DBS IPGs are associated with numerous clinical challenges and are prone to various complications. Advances in DBS IPG engineering constitute one of the most promising areas of growth in the field of functional neurosurgery. With the development of further insights into effective programming, together with novel hardware materials, IPG longevity may be extended. This may in turn result in reduced costs and complications associated with DBS therapy. In addition, the utilization of enhanced wireless recharging techniques may increase the practicality of the current devices. Novel external neuromodulation strategies may allow IPGs to become extracorporeal in the future.

## Author Contributions

Concept and design: CS, CI-M, MP, and SK. Supervision: SK. Literature search: CS, CI-M, and DA-P, AN. Writing manuscript: CS, CI-M, DA-P, AN, AF, and AZ. Figure and table design: CS, KY, and AZ. Critical review: NL, GI, CH, MH, AL, RM, AF, and SK. All authors contributed to the article and approved the submitted version.

## Conflict of Interest

AL has consulted for Medtronic, Abbott, Boston Scientific, Insightec, Aleva and is a co-founder of Functional Neuromodulation. SK received consulting fees from Medtronic. CS has received fellowship grants from Michael and Amira Dan Foundation and Turkish Neurosurgical Society. CI-M is founder and CEO of Hyperexis and Abaxial Médical Inc. AF reports the following: consultancies from Abbvie, Medtronic, Boston Scientific, Sunovion, Chiesi farmaceutici, UCB, Ipsen; Advisory Boards of Abbvie, Boston Scientific, Ipsen; honoraria from Abbvie, Medtronic, Boston Scientific, Sunovion, Chiesi farmaceutici, UCB, Ipsen; grants from University of Toronto, Weston foundation, Abbvie, Medtronic, Boston Scientific. RM is in the advisory board of Medtronic and receives grants from Medtronic. The remaining authors declare that the research was conducted in the absence of any commercial or financial relationships that could be construed as a potential conflict of interest.

## Publisher’s Note

All claims expressed in this article are solely those of the authors and do not necessarily represent those of their affiliated organizations, or those of the publisher, the editors and the reviewers. Any product that may be evaluated in this article, or claim that may be made by its manufacturer, is not guaranteed or endorsed by the publisher.
